# 3D Microstructure Simulation of Reactive Aggregate in Concrete from 2D Images as the Basis for ASR Simulation

**DOI:** 10.3390/ma14112908

**Published:** 2021-05-28

**Authors:** Xiujiao Qiu, Jiayi Chen, Maxim Deprez, Veerle Cnudde, Guang Ye, Geert De Schutter

**Affiliations:** 1 Department of Structural Engineering and Building Materials, Ghent University, 9052 Ghent, Belgium; Geert.DeSchutter@UGent.be; 2 Department of Materials, Mechanics, Management & Design, Delft University of Technology, 2600 AA Delft, The Netherlands; J.Chen-2@tudelft.nl (J.C.); G.Ye@tudelft.nl (G.Y.); 3 PProGRess/UGCT, Geology Department, Ghent University, 9000 Ghent, Belgium; Maxim.Deprez@UGent.be (M.D.); Veerle.Cnudde@UGent.be (V.C.); 4 Environmental Hydrogeology Group, Department of Earth Sciences, Utrecht University, 3584 CS Utrecht, The Netherlands

**Keywords:** reactive silica, aggregate microstructure, image analysis, gaussian filtering method, 3D simulation

## Abstract

The microstructure of alkali-reactive aggregates, especially the spatial distribution of the pore and reactive silica phase, plays a significant role in the process of the alkali silica reaction (ASR) in concrete, as it determines not only the reaction front of ASR but also the localization of the produced expansive product from where the cracking begins. However, the microstructure of the aggregate was either simplified or neglected in the current ASR simulation models. Due to the various particle sizes and heterogeneous distribution of the reactive silica in the aggregate, it is difficult to obtain a representative microstructure at a desired voxel size by using non-destructive computed tomography (CT) or focused ion beam milling combined with scanning electron microscopy (FIB-SEM). In order to fill this gap, this paper proposed a model that simulates the microstructures of the alkali-reactive aggregate based on 2D images. Five representative 3D microstructures with different pore and quartz fractions were simulated from SEM images. The simulated fraction, scattering density, as well as the autocorrelation function (ACF) of pore and quartz agreed well with the original ones. A 40×40×40 mm${}^{3}$ concrete cube with irregular coarse aggregates was then simulated with the aggregate assembled by the five representative microstructures. The average pore (at microscale μm) and quartz fractions of the cube matched well with the X-ray diffraction (XRD) and Mercury intrusion porosimetry (MIP) results. The simulated microstructures can be used as a basis for simulation of the chemical reaction of ASR at a microscale.

## 1. Introduction

The cracking caused by internal chemical reactions, such as delayed etrringite formation and alkali silica reaction (ASR), is one of the major durability problems in concrete structures [1,2,3]. ASR has been viewed as a ‘cancer’ in concrete structures since it was firstly discovered and named by Stanton in the 1940s [4]. ASR starts from the dissolution of the reactive silica in the aggregate under the attack of hydroxide ions that come from the pore solution in the cement paste [5]. The dissolved silica ions either react with the diffused alkali ions inside the aggregate or diffuse into the cement paste and then react with the alkali ions. Where the products will be formed depends on a lot of factors. According to the research of [6,7,8,9,10], the mineralogical nature of the aggregate greatly affects the cracking pattern of the ASR-affected concrete. According to the study of Ponce and Batic [6], for rapid-reacting aggregates containing amorphous silica such as opal and vitreous volcanic rocks, ASR product formation and expansion occur at the paste-aggregate interface transition zone (ITZ). However, for slow-reacting aggregates containing defected crystal quartz, such as siliceous limestone, gel occurrence and expansion come mainly from the inside of the aggregate. In other words, the microstructure of the reactive aggregate partially determines the initial expansion sites caused by ASR in the concrete.

Developing predictive models is important since it is able to give suggestions about ASR prevention for the to-be-built structure or maintenance measures for the ASR-damaged structures. In the present predictive mechanical models, several models have considered the spatial reaction and initial expansion sites. The models of [11,12] simulated the mechanical degradation of the concrete induced by ASR. The reaction and expansion sites were represented by several expansive points or gel pockets distributed randomly in the aggregate or in both the aggregate and the ITZ. A recent mesoscopic model [13] has explored the influence of the initial expansion sites on the ASR cracking patterns. The expansion sites are assumed to be a reaction rim on the surface of the aggregate or to be some gel pockets distributed randomly inside sphere aggregates. These models are able to provide acceptable predictions about the cracking propagation or the structural performance at a mesoscale (mm) or macroscale (m), even though the expansion sites are simplified. However, the chemical reaction mechanism, such as when and where ASR products begin to grow, how the expansion sites evolve, the influence of the mineralogy of the aggregate on the expansion sites, and so forth cannot be captured or explored by these models. In other words, the real microstructure of the aggregate is needed if one tries to simulate ASR as realistically as possible or if one tries to build a model that can drive the physical response from the chemical reaction.

Our project is to build such a realistic model. The first problem is how to obtain the 3D microstructure of the aggregate. The most regular computed tomography (CT) or focused ion beam milling combined with scanning electron microscopy (FIB-SEM) is a direct method to obtain the 3D microstructure of a porous material [14]. However, unlike cement paste, whose representative elementary volume (REV) is around 100×100×100 μm${}^{3}$, the REV of a coarse aggregate is usually at a mesoscale (mm) [15]. Such a REV can be imaged with CT; however, it is presently challenging to resolve the pores and silica particles with sizes below 1 μm with this technique. With FIB-SEM, the required spatial voxel size can be met; however, the sample size of several micrometers is too small to be representative of the heterogeneous aggregates. Additionally, applying both of these methods to characterize the aggregates can be done [16]. Yet, such a workflow is time-consuming and expensive, as specific equipment with limited availability is required.

Alternatively, the 3D microstructure can be derived by simulating it based on the information from 2D images. This approach could deliver 3D information in a fast and inexpensive manner. A number of simulation approaches have been developed in the past [17], and among them, the autocorrelation function method and simulated annealing method [18] were extensively examined. The autocorrelation function method has been used and extended by many researchers [19,20,21] to simulate the 3D representative microstructure of materials. This method firstly generates Gaussian population noises in a domain and then filters the noises based on the 2D autocorrelation function to match the phase volume fractions and surface-area fractions on the 2D images.

Based on their experiences [20,22], the Gaussian filtering method and image analysis are utilized in this paper to simulate the 3D microstructure (mainly the spatial distribution of pore and reactive silica) of an alkali-reactive siliceous limestone from Belgium from 2D images. Instead of finding a REV for the aggregate at a mesoscale, five separated representative microstructures (each one with a size of 100×100×100 μm${}^{3}$) with different pore fraction and reactive silica fraction following a true distribution in 2D images were simulated and upscaled to compose the microstructure of an irregular coarse aggregate in a simulated 40×40×40 mm${}^{3}$ concrete cube. The simulated microstructure of the limestone is compared visually (phase distribution characteristics), numerically (ACF, particle size distribution), and experimentally (porosity, silica fraction) with the original 2D images, and X-ray diffraction (XRD) and mercury intrusion porosimetry (MIP) results.

## 2. Research Significance

A better understanding of the influence of the aggregate microstructure on ASR is essential to develop the materials’ science-based design procedure for the newly-built concrete to prevent ASR. This study is an effort in that direction, where a 3D aggregate microstructure is simulated from 2D images providing an advantage to overcome the resolution limitation of a CT scan or FIB-SEM. The proposed method can be used by the ASR modelers to consider the realistic microstructure of the aggregate instead of assuming the aggregate to be a homogeneous sphere so that the fundamental mechanism can be simulated more realistically. Moreover, the method can be extended to other porous materials rather than aggregates. For example, the microstructure of cement paste can be reconstructed directly based on one representative 2D image.

## 3. Material and Experimental Methods

An alkali-reactive siliceous limestone from Belgium, which has been reported to cause damage to several concrete structures, such as bridges and dams [23], is used as the simulation object in the paper. Physical properties were determined firstly to distinguish the aggregate.

A 3D microstructure simulation is mainly based on the utilization of 2D quantitative information which can only be calculated from 2D microstructure images with a clear outline between particles. Based on this principle, optical petrography micrographs were firstly taken from thin sections of the limestone to identify its mineral compositions and their particle size. According to the thin section results, most of the minerals have a cryptocrystalline size (below 4 μm), which cannot be segmented by the Leica DMLP petrographic microscope (Leica, Wetzlar, Germany) with a maximum resolution of 200 mm in the laboratory. Thus, a scanning electron microscope (SEM) under a backscattered (BSE) mode was chosen to obtain the 2D microstructure (JEOL, Tokyo, Japan) of the aggregate with a clear outline between particles.

The simulated representative microstructures of the aggregate are used to assemble the aggregate embedded in mortar in a simulated concrete cube. XRD is then carried out, based on the thin section results that all of the minerals in the aggregate are crystalline, to obtain the mass composition of the minerals. The results are used to compare with the simulated average mineral fractions at a concrete scale.

For the porosity, simulated results are compared with MIP results. Other methods such as helium pycnometry and water absorption can be used to obtain the porosity of a porous material. However, water absorption has low liability, especially when the permeability of the material is low, such as the coarse aggregate, while Helium pycnometry results contain great errors [24].

### 3.1. Materials

The particle size of the siliceous limestone is between 4 mm and 20 mm. A bulk sample of about 13 kg was shipped by the producer to the lab. The different useful densities, the water absorption after 24 h, and the specific gravity were determined following the BS EN 1097-6 [25] and are given in Table 1. Test samples are subsampled from the bulk sample according to BS EN 932-1 [26] and BS EN 932-2 [27]. The specific gravity is the ratio of the surface-dried saturation density of the aggregate to water density. After determining the physical properties, the samples were stored at 105 ${}^{{}^{\circ}}$C until a constant weight (around 24 h) was maintained. Afterwards, the samples were kept under vacuum until the time of the MIP test. Razafinjato et al. [28,29] investigated the high-temperature behaviour of a wide range of siliceous and calcareous aggregates used in concrete. According to their results, siliceous limestone should be relatively thermally stable below 105 ${}^{{}^{\circ}}$C. This means that their thermal expansion does not exceed 0.25% and no micro-cracks were found on SEM images from samples heated to this temperature. Moreover, the decarbonation phase degree of calcite and transformation phase degree of quartz, which are the main compositions of the limestone, are both above 500 ${}^{{}^{\circ}}$C. Therefore, temperatures up to 500 ${}^{{}^{\circ}}$C should not have an effect on the aggregates.

### 3.2. Methods

#### 3.2.1. Optical Petrography

Thin section petrography was used to determine the mineral constituents in the limestone. A representative test sample of around 1 kg was obtained from the bulk sample conforming to BS EN 932-2 [27]. The test sample was subsequently dried at 40 ${}^{{}^{\circ}}$C until a constant weight was reached. Lastly, the test sample was impregnated with epoxy resin. Then, six standard petrographic thin Sections 28 × 48 mm${}^{2}$ were prepared from the test sample. Thin sections were made in such a way as to guarantee the representative that each section contains at least 20 aggregates with different size distributions. Thin sections were studied in a Leica DMLP petrographic microscope (Leica, Wetzlar, Germany) in transmitted plane polarized light (PPL) and crossed polarized light (XPL) using 5×–10×–20×–50× objectives. Micrographs were acquired using the Leica digital camera (Leica, Wetzlar, Germany) at a 2560 × 1920 resolution.

#### 3.2.2. Scanning Electron Microscope

In order to cover the diversity of the aggregate, five polished sections were prepared with about 40 differently sized aggregates included. The aggregate was taken from the bulk sample according to BS EN 932-2 [27]. BSE images were acquired using a JEOL scanning electron microscope (JEOL, Tokyo, Japan) equipped with an energy-dispersive X-ray (EDX) spectroscopy detector (JEOL, Tokyo, Japan) operated at 15 kV with three magnifications of 800×–1000×–2000× (with image resolution of 1280×960 pixels) and a Quanta FEC 450 scanning electron microscope operated at 20 kV with a magnification of 2500× (with image resolution of 2048×1770 pixels).

#### 3.2.3. X-ray Diffraction

Three representative aggregate samples (each around 100 g) were prepared from the bulk sample according to BS EN 932-2 [27] and then were ground using a grinding machine prior to chemical analysis. Around 9 g was then taken as the test sample from each ground sample. We also added 10 wt% (1 g) Zincite (ZnO) into each test sample. Measurements were carried out by using a Thermo-Fisher-Scientific ARL X’tra diffractometer (Thermo-Fisher-Scientific, Waltham, MA, USA) equipped with a Peltier cooled detector (Thermo-Fisher-Scientific, Waltham, MA, USA), operated at 40 kV and 35 mA using CuKα, from 5${}^{\circ }$ to $75^{{}^{\circ}}$ 2θ at 0.02 2θ increments with 1 s counting time increment. The X-ray wavelength was 1.54 A.

#### 3.2.4. Mercury Intrusion Porosimetry

MIP measurements were undertaken to investigate the pore size distribution of the aggregate. In order to contain the diversity of the pore distribution, six aggregates with a size of around 4 mm were taken from the sample kept under vacuum. For each measurement, around 1.3–2.3 g of the sample was used. Intrusion and extrusion were done in a low-pressure and high-pressure PASCAL 140 Series instrument (Thermo-Fisher-Scientific, Waltham, MA, USA). First, the sample was tested in the low-pressure instrument, where the pressure was gradually increased from 0 to 200 kPa and followed by an extrusion phase, during which the pressure was decreased to 100 kPa. Then, the sample was moved to the high-pressure instrument, where the sample was subjected to mercury pressures ranging from 0.1 to 200 MPa and back to 0.1 MPa. As such, the sample went again through an intrusion and an extrusion phase. The pore size distribution data were computed based on the Washburn equation [30]. The surface tension of mercury is 0.485 N/m${}^{2}$, and the contact angle of mercury with a limestone surface is $130^{{}^{\circ}}$.

## 4. Framework Development of 3D Microstructure Simulation

The microstructure simulation process can be divided into three parts. Firstly, the petrographic characteristics of the limestone and its 2D microstructure from SEM-BSE images were described in Section 4.1. The image threshold and binarization process were then elaborated in Section 4.2. Finally, Section 4.3 illustrates how to simulate the 3D microstructure based on the 2D data.

### 4.1. Thin Section Petrography and the 2D Microstructure Features from SEM-BSE

**Figure 1:**
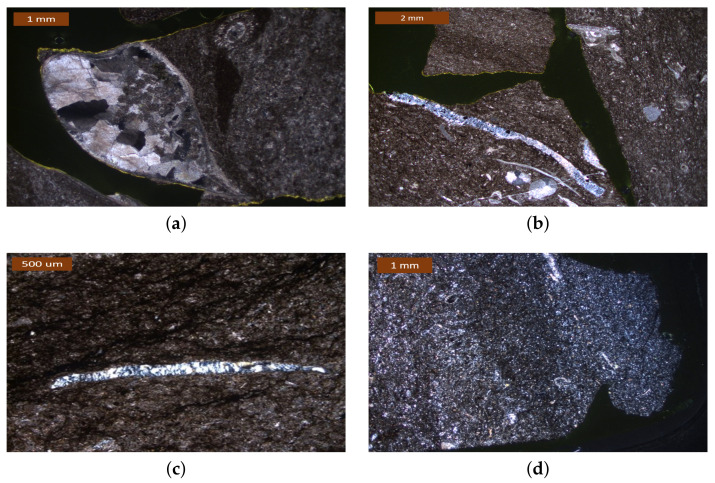
Optical micrographs of the siliceous limestone. (**a**) Well-crystallized sparite (white to black part) and micrite matrix under (dark-brown part) (XPL); (**b**) Partly micro- to crypto-crystalline quartz (blue and shining part) replaced in a shell in a micrite matrix under a magnification of 20× (XPL); (**c**) Micro- to crypto-crystalline quartz in a vein under a magnification of 10× (XPL) (**d**) Conspicuously distributed micro- to crypto-crystalline quartz under a magnification of 10× (XPL).

The minerals composing the limestone were identified based on their optical properties, as illustrated in [31]. The limestone is composed of micrite (microcrystalline calcite (CaCO${}_{3}$) with a crystal size less than 5 μm) with minor dolomite and interspersed microcrystalline and cryptocrystalline quartz (SiO${}_{2}$), as shown in Figure 1. Quantitative results (Section 5.3.1) show that the mass contents of calcite and quartz in this limestone are around 78.7% and 13.21%, respectively.

The rock also contains bioclastic fragments (fossil relics) filled or partly replaced with micro- to crypto-crystalline quartz (Figure 1b). The micro- to crypto-crystalline quartz occurs in some domain conspicuously dispersed throughout the carbonate (Figure 1d), partly also clustered in a vein (Figure 1c). A small amount of well-crystallized sparite (calcite crystals of over 5 μm) was also discovered, as shown in Figure 1a. According to [32], the alkali reactivity of this limestone mainly attributes to microcrystalline to cryptocrystalline quartz.

**Figure 2:**
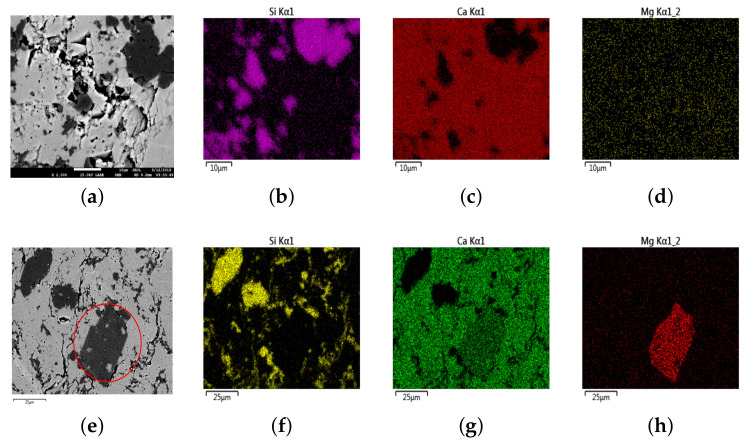
Two SEM-BSE images combined with EDX images show the mineralogical variability within two randomly selected areas on a thin section of the siliceous limestone. (**a**,**e**) SEM-BSE images at randomly selected areas; (**b**,**f**) EDX images of element Si; (**c**,**g**) EDX images of element Ca; (**d**,**h**) EDX images of element Mg.

Two-dimensional images with a clear outline between the particles are the prerequisite for 3D microstructure simulation. However, the Leica DMLP petrographic microscope (Leica, Wetzlar, Germany) in the laboratory is limited for this purpose, due to the pixel size limitation (200×200 μm${}^{2}$ under a magnification of 50×). Ever since the pioneering work by Scrivener and Pratt [33], BSE imaging has been widely used as a technique to examine the microstructure of cement-based materials [34,35]. In this study, we extended it to explore the microstructure of the coarse aggregate. Two representative SEM-BSE images with three energy-dispersive X-ray spectroscopy (EDX) analyses are shown in Figure 2. In a SEM-BSE image, phases with a higher atomic number can scatter more electrons so that they are brighter, while phases of a lower atomic number absorb more electrons and are darker. The brightness of a pixel is represented by its grey value on the SEM-BSE image. The higher the grey value is, the brighter the pixel. The grey value is positively related to the backscattering coefficient η, which is the fraction of scattered electrons. Based on this principle, in order to identify different phases on the BSE image, we calculated the backscattering coefficients for the minerals that were discovered by XRD, namely calcite, dolomite, fluorite, and quartz. The calculated coefficients η for them are 0.161, 0.143, 0.186, and 0.147, respectively [36].

Based on the calculated backscattering coefficients and the EDX images, in Figure 2a, the dark-grey part is quartz, and the black part is pore, while the light-grey part is calcite. Additionally, dolomite (CaMg(CO${}_{3}$)${}_{2}$) (red circle in Figure 2e) shows a similar dark-grey color to quartz, as their backscattering coefficient is very close to each other (0.143 and 0.147). However, it was found that in this siliceous limestone, the dolomite shows a cuboid crystal shape (red circle in Figure 2e), while the shape of the quartz crystal is relatively irregular. By checking the crystal grains visually, most quartz grains have a size between 1 μm and 10 μm, with part of the quartz crystals clustered into larger grains. The SEM-BSE images combined with EDX analysis confirms the presence of micrite and micro- to crypto-crystalline quartz and their distribution pattern, as analyzed from the thin section results. As stated previously that the main purpose of this work was to obtain the spatial distribution of the ASR-related phases (pore and reactive silica) for further ASR simulation, we only segmented quartz and pores from the background, as will be explained in the next section. The unreactive minerals (mainly of calcite and dolomite) were viewed as one single component, and are called "the others" in the continuation of this paper.

### 4.2. Thresholding & Binarization

A high-quality original SEM-BSE image is the prerequisite for accurate segmentation of features and subsequent quantification steps. More than 200 SEM-BSE images were taken under the magnification of 1000×, 2000×, and 2500×. However, as described above, the similar grey value between dolomite and quartz (Figure 2e) makes it difficult to segment quartz from dolomite. In order to avoid this issue, those SEM-BSE images that only contain quartz, pore, and calcite were selected based on the shape of the particles and the grey value, combined with EDX images. In the end, 90 SEM-BSE images were selected for binarization. Only after binarization, the pore area fraction and quartz area fraction can be calculated and their fraction distribution patterns can be analyzed. The area fraction is viewed as the volume fraction based on the ‘Delesse principle’ [37], and both are referred to as a fraction in the continuation of this paper. For optimum performance of binarization, images were firstly pre-processed to remove background noise through a median filter [38]. Each output pixel contains the median value in a two-by-two neighborhood around the corresponding pixel in the input image.

Image binarization means converting a grey-scale image with pixel values ranging from 0 (black) to 255 (white) in a 8-bit image into a binary image with a pixel value of either 0 (black) or 1 (white). The image will be correspondingly separated into two phases by comparing each pixel’s grey value to a grey-scale threshold. Binarization methods can be distinguished by the applied method to determine the threshold. Each method has their own equation and optimal parameters, as compared and concluded by Kim [39].

**Figure 3:**
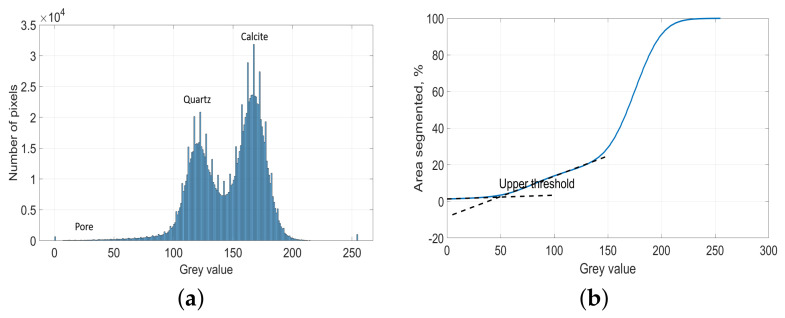
Grey value histogram of a SEM-BSE image and the corresponding method illustration to determine the grey scale threshold for the pore. (**a**) Grey value histogram of a SEM-BSE image with a low pore fraction of 0.6%; (**b**) Application of the overflow criteria to determine an upper threshold level for the pore. Dashed lines are the two linear curves around a grey value of 50. The grey value of the intersection point is the determined upper grey value threshold for the pore.

**Figure 4:**
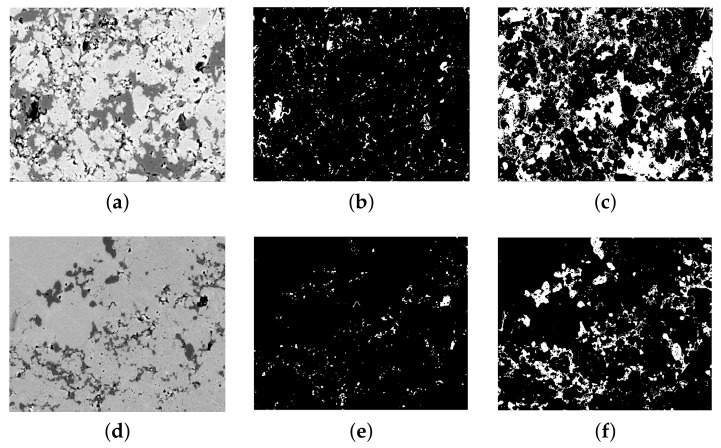
Two original SEM-BSE images and their binarized images of pore and quartz. (**a**,**d**) Original SEM-BSE images with low and high fractions of pore and quartz (dark is pore and dark-grey is quartz); (**b**,**e**) the pore distribution after binarization of Images 1 and 2, respectively. (**c**,**f**) The quartz distribution after binarization of Images 1 and 2, respectively. White is the pore in (**b**,**e**) and quartz in (**c**,**f**).

Due to the low pore fraction of the limestone, a separate pore peak does not occur in the grey-scale histogram of most of images, as seen in Figure 3a. Thus, the overflow segmentation method of Wong [40] was used in order to segment pores accurately. This method calculates the inflection point (the intersection between two linear segments, as shown in Figure 3b) of the cumulative area segmented curve as the upper grey-scale threshold, where a small incremental grey value will cause a sudden increase of pore fraction. The grey-scale threshold of quartz was calculated using Ostu’s standard method [41], since there is a strong contrast between quartz and the background. This method utilizes the zeroth- and the first-order cumulative moments of the grey level histogram and the discriminant analysis to calculate the threshold so that the intraclass variance of the thresholded black and white pixels is minimized. After thresholding, images were binarized for the pore and quartz, repetitively. The fractions of pore, quartz, and the others were then calculated from the binarized images. Figure 4 shows two original images with its binarization images of pore and quartz. It can be visually seen that the location, particle size, particle distribution of the pore, and the micro- to crypto-crystalline quartz have been separated from the background very successfully using the above segmentation methods.

**Figure 5:**
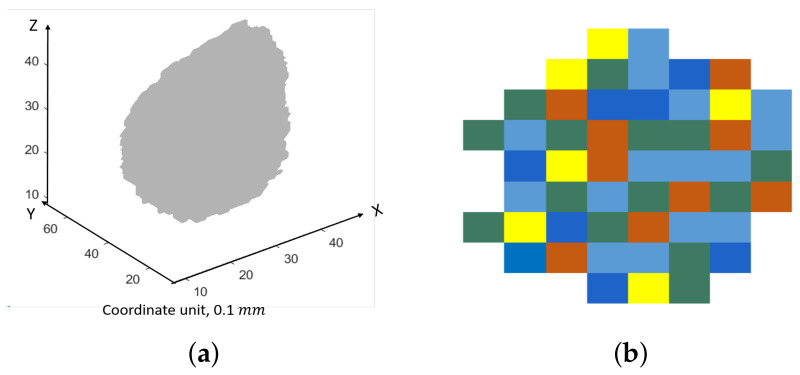
Schematic diagram of the method to simulate the microstructure of an irregular coarse aggregate. (**a**) A real 3D coarse aggregate. The coordinate unit is 0.1 mm. (**b**) One random slice of the microstructure of the coarse aggregate, which is constituted by a few representative microstructures. Different colors represent different representative microstructures.

As stated in Section 1, the REV of the coarse aggregate used in concrete is usually bigger than 1×1×1 mm${}^{3}$ [15]. If we want to simulate the real 3D microstructure of the siliceous limestone in such a relatively large size, a 2D image with a size of at least 1×1 mm${}^{2}$ would be needed. However, the microstructural information, such as the micro- to crypto- crystals, will not be captured in such a large image considering the pixel size limitation. Instead of trying to obtain such a large REV, we simulated a few separated representative 3D microstructures (a size of 100×100×100 μm${}^{3}$) from 2D images at a microscale so that the real microstructure information at a microscale from 2D SEM-BSE images can be captured as much as possible. These representative 3D microstructures at a microscale (μm) are then used to assemble the real aggregate in concrete at a mesoscale (mm). For more clarity, a brief schematic diagram of this method is shown in Figure 5. Figure 5a shows a real 3D irregular coarse aggregate with a size of 4 mm. Figure 5b illustrates a random representative 2D slice of its microstructure which is composed by a few representative microstructures, as represented by different colors. An application of this method and the upscaling process is further detailed in Section 5.3.2. With this method, a REV of the aggregate is avoided.

We set the simulation voxel size at a microscale as 1×1×1 μm${}^{3}$, considering the quartz particles are almost all above 1 μm. The minimum pore size was not considered because it can be as small as a few nanometers, which would cause a big burden on the computer. More random-access memory would be needed and the simulation would be very slow if the voxel size is set to be a few nanometers. In order to match the simulation voxel size, the original SEM-BSE images with a size of 1280×960 pixels (0.1×0.1 μm${}^{2}$/pixel) were then resized to a size of 128×96 pixels with a pixel size of 1×1 μm${}^{2}$ and binarized again. The influence of resizing on the pore and quartz fraction and distribution is discussed in the next paragraph. Resizing was realized using the bicubic interpolation method [42] that the output pixel value is a weighted average of pixels in the nearest 4-by-4 neighborhood. One can also skip the resizing step, but must be aware that more computation resources will be needed if the pixel size is too small.

**Figure 6:**
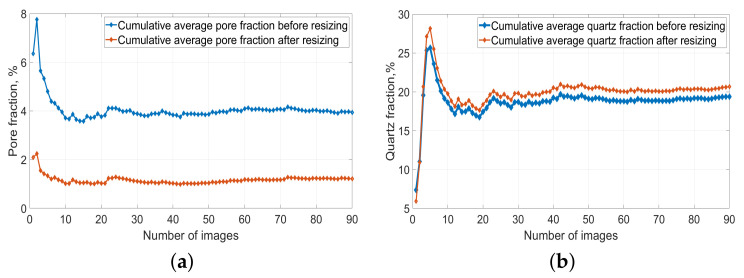
Cumulative average fraction of pore and quartz within 90 binarized images before and after image resizing. (**a**) Cumulative average pore fraction before and after image resizing; (**b**) Cumulative average quartz fraction before and after image resizing. The x-axis is the number of images used to calculate the cumulative average pore or quartz fraction. The y-axis is the cumulative average pore or quartz fraction.

In order to investigate the influence of resizing on the mineral fraction, we calculated the pore and quartz fraction for each binarized image and the cumulative average fraction for pore and quartz before and after resizing, respectively, using all of the 90 binarized images. Figure 6 shows the results. The blue curve in Figure 6a shows that the average pore fraction of the limestone is around 4% before resizing, while the red curve shows that it reduced to around 1% after resizing. Figure 6b shows that the average quartz fraction before resizing is around 19% and becomes 21% after resizing. It can be seen that after resizing, the pore fraction was greatly reduced by more than 50%, while the quartz fraction only fluctuated in a small range (within 2%).

**Figure 7:**
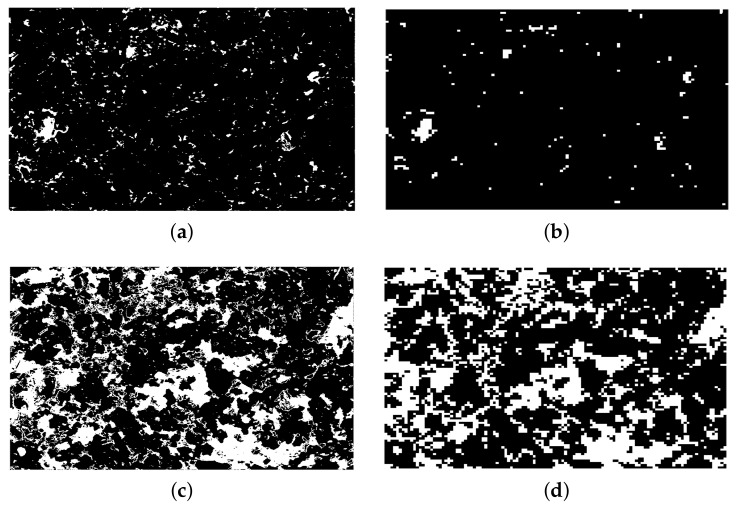
Pore and quartz distribution of the original SEM-BSE image shown in Figure 4a before and after image resizing. (**a**) Pore distribution before resizing; (**b**) pore distribution after resizing; (**c**) quartz distribution before resizing; (**d**) quartz distribution after resizing.

Figure 6 also shows that the minimum number of images required to give a stable average fraction is around 30 for both pore and quartz. Figure 7 shows the 2D pore and quartz distribution change after resizing of the original SEM-BSE image in Figure 4a. It can be concluded that after resizing, the pore distribution becomes much sparser, while the quartz particle distribution almost remains the same, except that a small part of the isolated particles are not withheld. This result indicates that the pore size in this limestone is at both a nanoscale and microscale, with most of the pores at a nanoscale, while the quartz crystal size is mostly at a microscale. What should be kept in mind is that the pore fraction to be simulated is actually the air void fraction (over 1 μm), since our simulation voxel size is 1×1×1 μm${}^{3}$.

### 4.3. 3D Simulation

#### 4.3.1. Representative Image Selection

**Figure 8:**
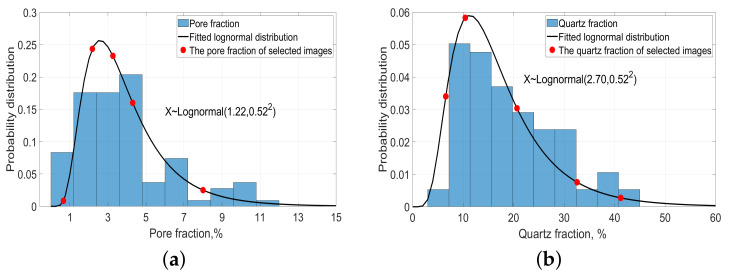
Normalized histograms of pore and quartz fractions based on 90 binarized images and the calculated lognormal probability density functions. (**a**) The normalized histogram and the lognormal probability density function of the pore fraction; (**b**) the normalized histogram and the lognormal probability density function of the quartz fraction. Red solid circles point to the pore or quartz fractions and their probability values of the selected SEM-BSE images for 3D simulation.

The distribution of minerals in the siliceous limestone is very heterogeneous, especially the quartz distribution. Therefore, representative 2D SEM-BSE images with the size of 128×96 μm${}^{2}$ should be selected carefully for 3D simulation. In our work, we assumed that the mineral fraction follows a lognormal distribution in reality. It is a convenient and useful model in engineering sciences [43]. If *x* has a normal distribution, then the exponential function of *x*, y=exp(x), has a lognormal distribution. A random variable which is lognormally distributed takes only positive real values. The representative images were then selected in the following way:

- Firstly, the normalized histograms of pore fraction and the quartz fraction from all of the 90 binarized images were drawn as shown in Figure 8. In Figure 8, the fraction distribution is divided into 10 bins represented by the blue bar. The x-axis is the pore or quartz fraction. The y-axis represents the probability distribution of the pore or quartz fraction within 90 binarized images.
- Based on the histogram, the lognormal distributions were fitted for the pore and quartz fractions, respectively. Equation (1) is the lognormal probability density function.
  (1) $$P=\frac{1}{X\sigma \sqrt{2\pi }}e^{\frac{-{\left(ln\left(X\right)-\mu \right)}^{2}}{2\sigma^{2}}},$$
  where *X* is the pore or quartz fraction, and *P* is the probability density at fraction *X*. μ is the mean value and σ is the standard deviation. These are the mean value and standard deviation of the variable’s natural logarithm, not the expectation and standard deviation of *X* itself. μ and σ can be calculated through μ= mean(ln(*X*)) and σ= std(ln(*X*)), respectively. The fitted (μσ) based on the data from 90 images for pore and quartz are (1.20, 0.50) and (2.70, 0.52), respectively. The functions are drawn in Figure 8 by a black curve.
  Three representative statistical variables (the mode, the mean, and the median with their definition shown below Table 2) of the pore and quartz fraction calculated from the fitted lognormal distribution and images are shown in Table 2. The differences of these variables of pore fractions between the fitted curve and original data from 2D images are very small, except the mode pore fraction of the fitted curve is around 1% lower than the original one. The differences of these variables of quartz fraction between the fitted curve and raw data are also small, mainly below 2%, except that the median quartz fraction of the fitted curve is 4% higher than that of the original data. Generally speaking, the fitted lognormal distribution of the pore and quartz fraction can represent the true distributions pore and quartz fractions in the selected 90 images.
- Finally, representative fractions were selected through the probability density function curve. In total, 10 representative images were selected: Five images with quartz fractions of (6.58%, 10.44%, 20.68%, 32.58%, 41.23%) and another five images with a pore fractions of (0.66%, 2.18%, 3.26%, 4.3%, and 8.0%) as indicated by the x-axis values of the red solid circles in Figure 8a,b, respectively. These five fractions are representative not only because they cover the whole fraction distribution span of silica or pores, but also capture the main characteristics of the fraction distribution, such as the global maximum fraction value.

**Figure 9:**
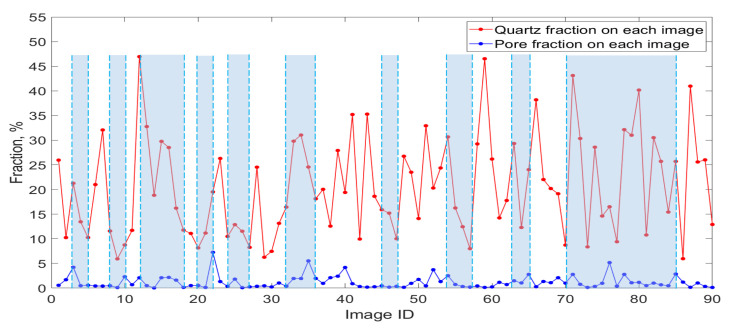
The pore and quartz faction distributions on 90 images. The light-blue shade is the positively correlated part, where the pore fraction increases or decreases accordingly as the quartz fraction increases or decreases.

Figure 9 shows the correlation between the pore and quartz fraction distribution on 90 images. It can be seen that except in parts of images where the pore fraction remains relatively stable, almost half of the images (indicated by the blue part, around 43 images) have a positive correlation between the pore and quartz fractions, which means that as the quartz fraction increases or decreases, the pore fraction increases or decreases correspondingly. Therefore, the selected pore and quartz fractions are positively correlated in the simulated 3D microstructures.

The final five simulated 3D microstructures (with microstructure ID from 1 to 5) have pore fractions and quartz fractions as follows: Microstructure 1: (0.11%, 6.48%); Microstructure 2: (0.51%, 10.41%); Microstructure 3: (0.93%, 20.51%); Microstructure 4: (1.45%, 32.42%); and Microstructure 5: (2.77%, 40.91%). Again, the simulated pore fraction is actually the air void fraction pore size over 1 μm.

#### 4.3.2. Simulation Method

According to Quiblier [44], a 2D image of a porous medium can be characterized by two functions: (1) A probability density function (PDF) or the histogram of an image; or (2) an autocorrelation function (ACF). The physical meaning of the ACF in this paper is a measure of the probability that two pixels on an image at a specific distance belong to a same phase. Most importantly, these two characteristics remain unchanged for two and three dimensions. It should be noted that the porous medium does not need to be isotropic. An anisotropic ACF can be obtained by analysing several mutually perpendicular thin sections or SEM images.

Let I(i,j) be the value 1 or 0 depending on whether the pixel P(i,j) is located in a phase s of interest or not. The probability function for an M×N image is determined using Equation (2) [22]:

(2) $$P\left(s\right)=\frac{\sum_{i,j}I\left(i,j\right)}{M\times N},$$

where *s* is the target phase, which is pore or quartz in this paper. The autocorrelation function of phase *s* is evaluated using the following Equation (3):

(3) $$S_{1}\left(x,y\right)=\sum_{i=1}^{M-x-1}\sum_{j=1}^{N-y+1}\frac{I(i,j)I(i+x-1,j+y-1)}{\left(M-x+1)(N-y+1\right)},$$

where *x*, *y* are the coordinate values of a pixel on the image, and 1≤x≤M,1≤y≤N. One may recognize that Equation (3) is a convolution where the kernel is rotated $180^{\circ }$, and as such, can be performed rapidly using Fourier transform methods to improve the calculation. Given the two-dimensional calculated $S_{1}\left(x,y\right)$, we can obtain the desired one-dimensional or isotropic autocorrelation function $S_{3}\left(r\right)$ by averaging over $S_{2}\left(r,\theta \right)$ values, whose value should be calculated through the Equation (4), at a fixed radius *r*, as shown by Equation (5).

(4) $$\begin{array}{c} S_{2}\left(r,\theta \right)=S_{1}\left(r\cos\theta ,r\sin\theta \right) \end{array}$$

(5) $$\begin{array}{c} S_{3}\left(r\right)=\frac{1}{2r+1}\sum_{i=0}^{2r}S_{2}\left(r,\frac{\pi l}{4r}\right), \end{array}$$

where the value $S_{1}\left(r\cos\theta ,r\sin\theta \right)$ can be calculated by bilinear interpolation from the value of $S_{1}\left(x,y\right)$.

After getting the one-dimensional autocorrelation function $S_{3}\left(r\right)$, the 3D microstructure reconstruction can be achieved by four steps.

Step 1: Generation of a random number N(x,y,z) at each point of the target domain following a normal distribution. All *N* points are completely uncorrelated. The initial phase at each point in this domain is considered as the others in our work.

Step 2: For each point (x,y,z), considering a neighbouring domain composed of points (x+i,y+j,z+k) up to a certain distance which corresponds to the number of steps beyond which ACF becomes negligible (10 μm for quartz and 5 μm for pores in this paper). The values *N* in this neighbourhood are then linearly combined so as to form a new number denoted as R(x,y,z), which is correlated and defined by Equation (6). The periodic boundary condition is applied in the *X*, *Y*, and *Z* directions during the calculation of *R* in our work.

(6) $$R\left(x,y,z\right)=\sum_{i,j,k}F\left(i,j,k\right)N\left(x+i,y+j,z+k\right)$$

It has been proven by Bentz [22] that the 3D ACF F(r) calculated from the 2D ACF $S_{3}\left(r\right)$ by Equation (7) can be expressed as a function of the coefficient F(x,y,z).

(7) $$F\left(r\right)=F\left(x,y,z\right)=\frac{S_{3}\left(r=\sqrt{x^{2}+y^{2}+z^{2}}\right)-S_{3}{\left(0\right)}^{2}}{S_{3}\left(0\right)-S_{3}{\left(0\right)}^{2}},$$

where $S_{3}\left(0\right)$ is the area fraction of the target phase on the image.

Step 3: A threshold *R* needs to be operated on the filtered domain to match the approximate 2D fraction of each phase of interest.

Step 4: Repeating Steps 1 to 3 until all the phases of interest are reconstructed.

According to Bentz [22], further modification may be required to adjust the 3D ACF through adjusting the hydraulic radius of the interested phase. However, in our method, this issue is solved in Step 2 by setting the neighbour range of each point to the 2D ACF “pessimum value”, and no further adjustment is needed.

## 5. Experimental Corroboration of the Simulated 3D Microstructure

**Figure 10:**
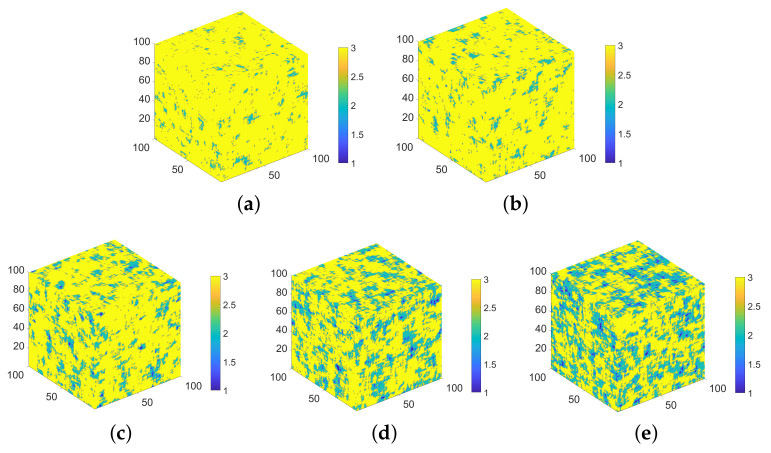
The final five simulated 3D microstructures of the siliceous limestone from 2D images. (**a**) Microstructure 1 with a pore and quartz fraction of (0.11%, 6.48%); (**b**) Microstructure 2 with a pore and quartz fraction of (0.51%, 10.41%); (**c**) Microstructure 3 with a pore and quartz fraction of (0.93%, 20.51%); (**d**) Microstructure 4 with a pore and quartz fraction of (1.45%, 32.42%); (**e**) Microstructure 5 with a pore and quartz fraction of (2.77%, 40.91%). There are three phases in all microstructures: 1-pore, 2-quartz, and 3-the others.

Five 100×100×100 μm${}^{3}$ microstructures at a microscale with a voxel size of 1×1×1 μm${}^{3}$ were simulated based on the methods described previously. Firstly, the 3D pore distribution with different fractions was simulated in each domain of 100×100×100 μm${}^{3}$ based on the five 2D selected representative images of pores. After that, the 3D quartz distribution with different fractions was simulated in each domain based on the five 2D selected representative images of quartz. A periodic boundary condition in the *X*, *Y*, and *Z* directions was used during the simulation. Figure 10 shows the final five simulated 3D microstructures. From Microstructures 1 to 5, the pore fraction increases from 0.11% to 2.77%, and the quartz fraction increases from 6.48% to 40.91%. The pore and quartz fraction is positively correlated in each simulated 3D microstructure, as described in Section 4.3.1. Once again, the simulated pore fraction is actually the air void fraction (pore size over 1 μm).

The simulated particle sizes and their distribution characteristics of pore and quartz were first checked visually by comparing the random 2D slices selected from the 3D simulated microstructures with an original 2D image. Numerical checking was subsequently completed so that the ACF calculated from the simulated microstructures was compared with the original ACF of the original 2D images. Then, the five microstructures were upscaled to simulate the irregular coarse aggregate embedded in a 40×40×40 mm${}^{3}$ concrete cube. The upscaling technique is detailed in Section 5.3.1. The average volume fraction of the quartz and air void fraction of the simulated aggregates in the concrete cube were compared with the quantitative results from XRD and MIP. The whole simulation work was implemented using MatLab-R2019b (R2019b, 2019, The Mathworks, Natick, MA, USA).

### 5.1. Visual Checking

**Figure 11:**
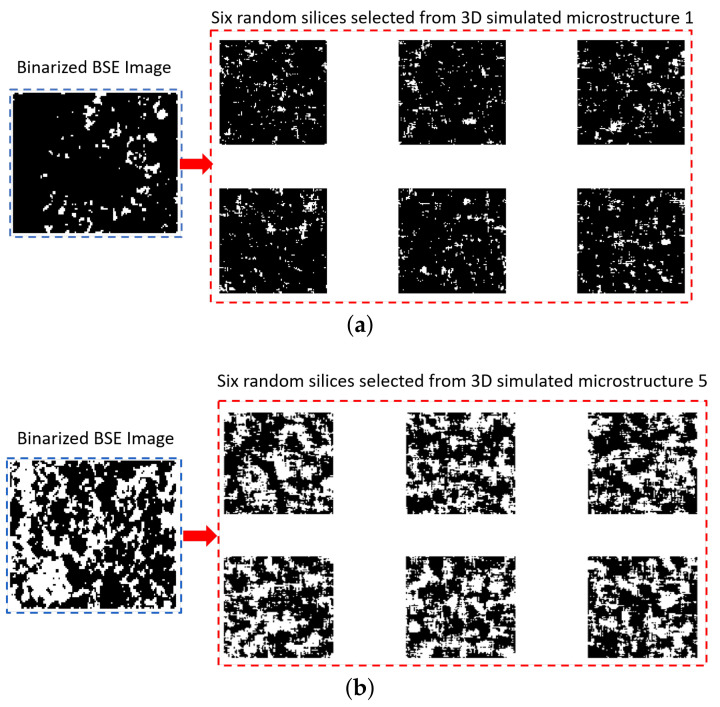
Two examples of the 2D original and simulated quartz distribution. (**a**) Comparison between six 2D sections from the simulated Microstructure 1 with 6.48% quartz fraction and the original 2D binarized image; (**b**) comparison between the 2D sections from the simulated Microstructure 5 with 40.91% quartz fraction and the original 2D binarized image. White: quartz; Black: pore and the others.

**Figure 12:**
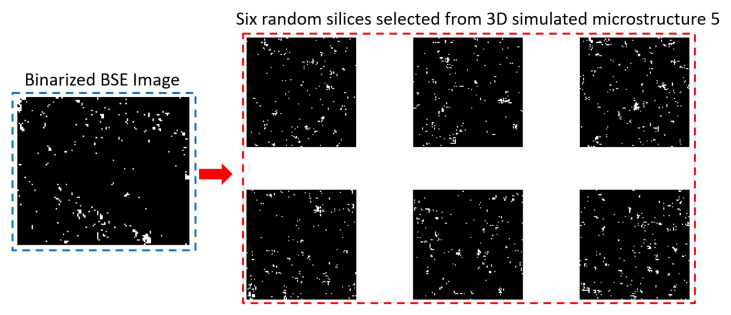
An example of a 2D pore distribution comparison between the simulated 2D sections from Microstructure 5 with a pore fraction of 2.77% and the original binarized image. White: pore; Black: quartz and the others.

Figure 11 shows six random slices of quartz distribution from the simulated 3D microstructure 1 (lowest quartz fraction) and 5 (highest quartz fraction) and their original binarized images. Figure 11a shows that at a lower quartz fraction, the quartz particle sizes are smaller and distributed sparsely in the simulated model, which is similar to the original image. For a higher quartz fraction in Figure 11b, quartz particles are clustered together, which makes the structure more closely connected. This characteristic is also simulated relatively successfully if one compares it with the original one. Figure 12 is an example of a 2D pore distribution comparison between the 2D sections from the simulated Microstructure 5 with the highest pore fraction of 2.77% and its original image. Due to the low pore fraction above 1 μm (average is around 1%, as shown by the red curve in Figure 6a) of the limestone and the resizing effect, the pore is scattered sparsely. The pore size and distribution pattern also looks similar to the original one. Therefore, to the naked eye, a similarity undoubtedly exists in the size and ‘scattering’ of the pore and quartz between the simulated microstructure and the real image.

### 5.2. Numerical Checking

The ACF curves of pore and quartz in the original 2D images are the input for the 3D microstructure simulation. In order to check if the simulation successfully remains as the characteristics of the original ACF, in this section we computed the ACF curves from the five simulated 3D microstructures and compared them to those of the input original images.

**Figure 13:**
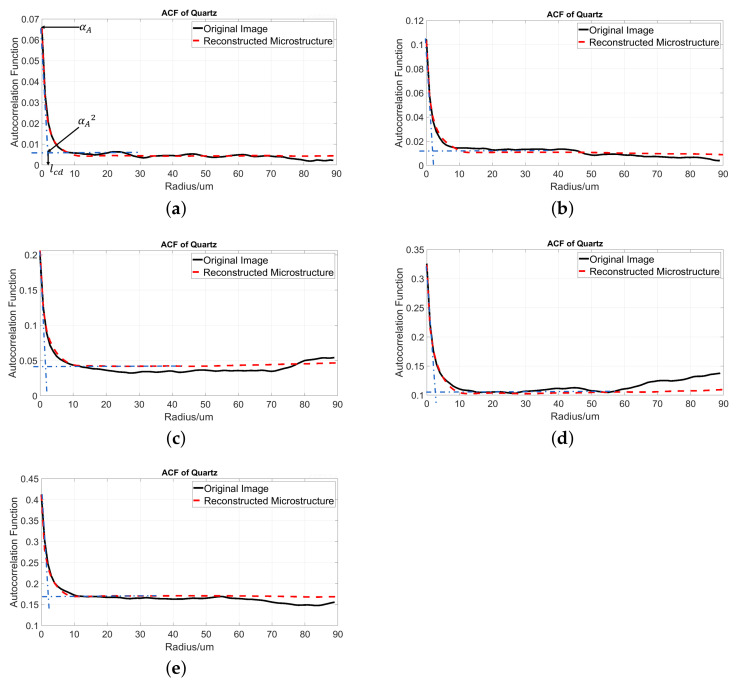
The ACF of quartz from 2D images (black curve) and 3D simulated microstructures (red dashed curve). (**a**) ACF from Microstructure 1 with quartz fraction of 6.48% and its original image; (**b**) ACF from Microstructure 2 with quartz fraction of 10.41% and its original image; (**c**) ACF from Microstructure 3 with quartz fraction of 20.51% and its original image; (**d**) ACF from Microstructure 4 with quartz fraction of 32.43% and its original image; (**e**) ACF from Microstructure 5 with quartz fraction of 40.91% and its original image; two dashed blue lines are the tangent line at r=0 and the plateaus line at where the quartz ACF becomes stable, respectively.

Figure 13 shows the ACF curve of quartz. The red dashed curve is the ACF from the simulated 3D microstructure, while the black curve is the ACF from the original 2D image. It is obvious that the original and reconstructed ACF are seen to nearly overlap in the distance range of a value from 0 to around 10 μm for quartz in all of the subfigures. As stated before, the ACF describes the probability of two random points with a distance *r* located in the same phase. The distance range of 0 to 10 μm is actually the PSD of quartz, and 10 μm is the maximum 2D particle size of quartz. Therefore, we can conclude that the simulated PSD agrees very well with the original PSD. At a longer distance, the curves do not overlap too well. The original ACF curve fluctuates due to the random nature, as also found in [22], while the simulated ACF curve is flat due to the extent of the filter operation used in the generation algorithm. However, this small difference can be ignored, since it does not influence the PSD.

Except the PSD, the ACF curve contains more information, such as the mineral fraction $\alpha_{A}$, the value ${\phi_{A}}^{2}$ when the ACF function becomes stable, the characteristic particle size $L_{cd}$, and the specific surface area $\alpha_{surf}$ (ratio of the phase surface area to the total volume of the sample) of the phase of interest [20,45].

Taking Figure 13a as an example, the value of the curve at r=0 corresponds to the 2D quartz fraction from the 2D image (black curve) and the 3D quartz fraction from the corresponding reconstructed microstructure (red dashed curve). The plateaus value of the ACF function corresponds to ${\phi_{A}}^{2}$. The intersection of the slope of ACF at r=0 and the plateau region indicates a characteristic dimension of the quartz structure $l_{cd}$. This parameter can be used to calculate the average quartz particle size $d_{av}$ according to $d_{av}=l_{cd}/\left(1-\alpha_{A}\right)$ [46]. The slope of the ACF at r=0 relates to the specific surface area $\alpha_{surf}$ of the quartz. By comparing the ACF curves in Figure 13, we can say that all the mentioned parameters of the simulated microstructures match very well with that of the original images, except Figure 13c, where the plateaus value of the original image is a little bit lower than the simulated one.

**Figure 14:**
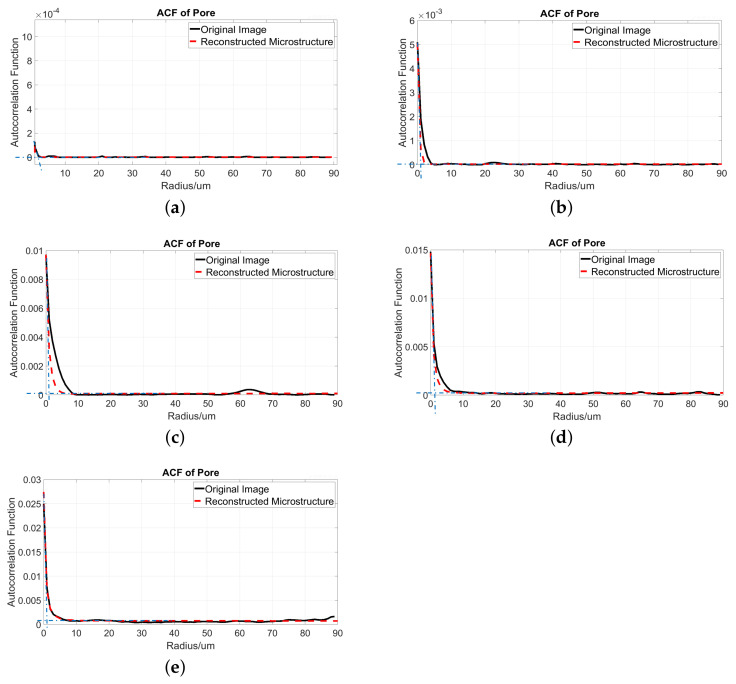
The ACFs of pores from 2D images (black curve) and 3D simulated microstructures (red dashed curve). (**a**) ACF from Microstructure 1 with a pore fraction of 0.11% and its original image; (**b**) ACF from Microstructure 2 with a pore fraction of 0.51% and its original image; (**c**) ACF from Microstructure 3 with a pore fraction of 0.93% and its original image; (**d**) ACF from Microstructure 4 with a pore fraction of 1.45% and its original image; (**e**) ACF from Microstructure 5 with a pore fraction of 2.77% and its original image; two dashed blue lines are the tangent line at r=0 and the plateaus line at where the pore ACF becomes stable, respectively.

Figure 14 shows the corresponding ACF curves of the pore. The simulated ACF and original ACF almost overlap in the distance range from 0 to 5 μm, except in Figure 14c with a pore fraction of 0.93%, where the simulated PSD is narrower than the original figure. However, the pore fraction, the slope at r=0 of the red dashed and black curves in each subfigure are the same, as well as the plateaus value. Generally, the same conclusion as for quartz can be made for pores, that the simulated ACF of pores agrees well with the original one.

### 5.3. Quartz Fraction from Model and XRD

#### 5.3.1. Quantitative Results from XRD

**Figure 15:**
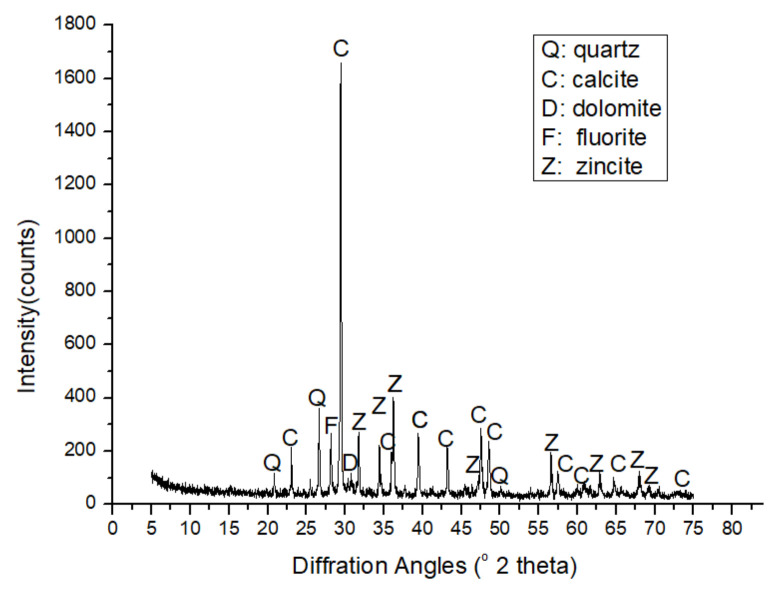
XRD pattern of the limestone.

The XRD pattern in Figure 15 confirms the observations from the thin section and SEM results that this limestone is composed mainly of calcite (volume fraction of 78.7%) and quartz (volume fraction of 13.21%) with a small part of dolomite and fluorite (volume fraction of 8.08%). The mass and volume fractions of the minerals are shown in Table 3. The volume fractions were calculated by dividing the mass fraction (obtained using the software TOPAS (Academic V4.1, 2007, Coelho Software, Brisbane, Australia) by the corresponding density. The density of calcite, dolomite, fluorite, and quartz is 2.71 g·cm${}^{-3}$, 2.84 g·cm${}^{-3}$, 3.13 g·cm${}^{-3}$ and 2.65 g·cm${}^{-3}$, respectively, as obtained from [47].

#### 5.3.2. Upscale from Microscale to Mesoscale

In order to compare the simulated mineral (pore, quartz, the others) volume fraction with the real volume fraction from XRD or MIP, a concrete cube of 40×40×40 mm${}^{3}$ was simulated. The size of 40×40×40 mm${}^{3}$ was chosen because the minimum size of a REV of concrete at a mesoscale should be at least 2.5 times bigger than the maximum aggregate size (16 mm in this paper) [48,49]. The cube is composed of mortar and irregular aggregate with a particle size from 4 mm to 16 mm. An aggregate volume fraction in concrete of 30% was used for simulation. The microstructure of the coarse aggregate was composed by the five representative simulated 3D microstructures. The simulation process and upscaling technique is described below.

Firstly, the voxel size of the concrete cube was set to be 100×100×100 μm${}^{3}$, which means that the cube size of the simulated 3D microstructure is now the voxel size at mesoscale. Therefore, the final simulated cube has a lattice size of 400×400×400.

**Figure 16:**
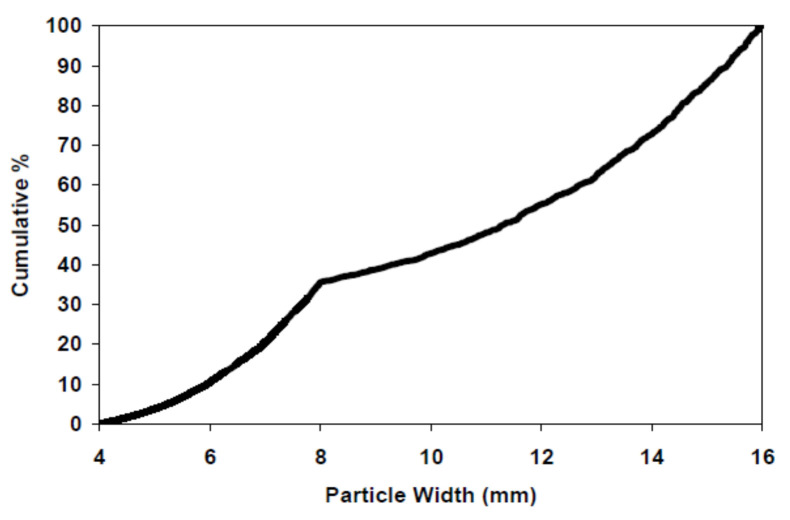
Particle-size distribution for the coarse aggregates in the concrete.

**Figure 17:**
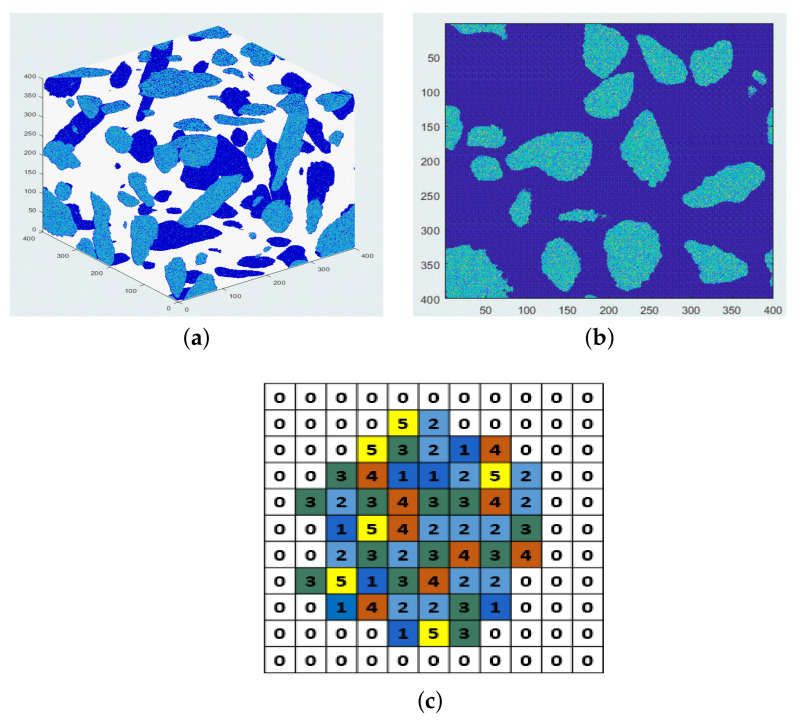
A simulated 40×40×40 mm${}^{3}$ concrete cube with a voxel size of 0.1×0.1×0.1 mm${}^{3}$. (**a**) 3D illustration of the cube surface; (**b**) a random 2D slice of the cube; (**c**) schematic diagram of the microstructure of one coarse aggregate. 0: mortar; 1–5: the five reconstructed 3D microstructures, respectively.

Then, the irregular aggregates from 4 mm to 16 mm were distributed randomly inside the cube using the ANM model of Qian [50]. The volume fraction of the aggregate at different sizes from 4 mm to 16 mm was calculated based on the cumulative distribution function (CDF) of the coarse aggregate, as shown in Figure 16. The surface of the simulated cube and a random slice of the simulated cube are shown in Figure 17a,b.

After this step, each node in the cube is 0 or 1, where 0 represents mortar and 1 represents the aggregate. By accounting the node number of 1 of the cube, we found that the total nodes occupied by aggregates were 19,136,000 and the simulated aggregate volume fraction in the cube was 29.9%, which is very close to the true volume fraction.

The last step was to allocate the five simulated 3D microstructures to these 19,136,000 nodes. Numbers 1 to 5 were used to represent the five simulated 3D microstructures, respectively, as shown in the first column in Table 4. The volume fraction occupied by each microstructure was calculated according to the fitted lognormal distribution function of the quartz fraction, as determined in Section 4.3.1 (Figure 8b, X∼Lognormal ($2.70,0.52^{2}$)). The calculated volume fraction of each microstructure was shown in the third column in Table 4. Then, the number of nodes for each microstructure was calculated to be $1.053\times 10^{6}$, $1.2967\times 10^{7}$, $3.862\times 10^{6}$, $7.90\times 10^{5}$ and $4.96\times 10^{5}$, respectively, as shown in the second column in Table 4. Subsequently, we randomly assigned $1.2967\times 10^{7}$ nodes out of node 1 to be 2 (microstructure 2) and repeated this process until each node 1 was reassigned. A schematic diagram of the final microstructure of one aggregate at mesoscale is shown in Figure 17c.

#### 5.3.3. Quartz Fraction Comparison

Table 4 also shows the simulated average mineral fractions (mineral volume/aggregate volume) of the concrete cube. The simulated average quartz fraction at mesoscale is around 13.93% (Table 4, Column 5). It only increased a factor of about 1.05 compared with the real quartz fraction (13.21%) obtained from XRD. Consequently, the fraction of the others reduced from 86.79% to 85.43%. It can be seen that the simulated compositions agrees very well with the XRD results only with a relative error less that 6% for both the quartz and the others.

### 5.4. Pore Fraction from Model, SEM and MIP

**Figure 18:**
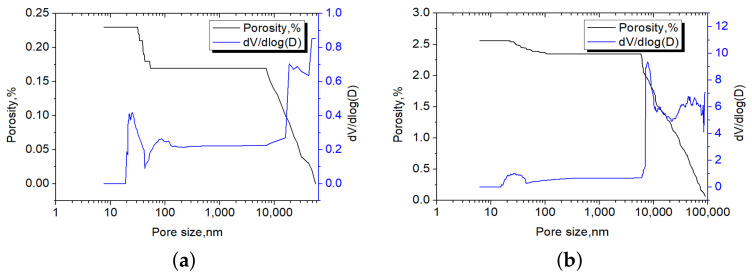
Pore size distribution of the siliceous limestone from MIP results. (**a**) Pore size distribution of a sample with porosity of 0.23%; (**b**) pore size distribution of a sample with porosity of 2.6%. Left y-axis is the porosity for the black curve. Right y-axis is the volume/pore-size for the blue curve.

Figure 18 shows two representative pore size distribution patterns of the limestone: a low pore fraction of 0.23% (Figure 18a) and a high pore fraction of 2.56% (Figure 18b) obtained from MIP. It can be seen from the blue curve in Figure 18 that the pore size from the MIP results is mainly located between 10 nm and 100 nm, and over 10 μm. Most of the pores are over 10 μm, while almost no pore between 100 nm and 10 μm was found.

One should remember that in our model, the simulated pore fraction only considers the aid void (pore size over 1 μm). Therefore, a pore fraction over 1 μm was extracted from the MIP results and concluded in Table 5. The results were then compared with our simulation result in Table 4. The data in the second column of Table 4 shows that the air void fraction in the simulated concrete cube mainly varies from 0.11% to 2.77%, which is close to the distribution range of 0.09% to 2.35% from the MIP results, as shown in Table 5 (column 3). Additionally, the simulated average air void fraction of the concrete cube (Table 4, column 4) is 0.65%, which also agrees well with that of MIP results (0.7%). Moreover, the average air void fraction obtained from SEM-BSE images was around 1% (the stable cumulative average value of the red curve in Figure 6a), where only 0.35% was lost during the simulation. The above analysis indicates that the simulated pore fraction distribution matches well with the results derived with MIP, and the simulated average pore fraction over 1 μm is also close to that of the MIP results, as well as of the SEM results. However, one should be aware that MIP may underestimate the true pore fraction because the mercury is unable to access all of the inner open pores or if less open pores’ frequencies are on the surface, while the image analysis could overestimate the true pore fraction since it is the 2D intersection of the 3D pore being analyzed, and the binarization process can induce error as well.

## 6. Application of the Model

The main purpose of obtaining the microstructure of the alkali-reactive aggregate is to use it as a basis in a ASR multiscale simulation model. Such a model starts the simulation of ASR from the chemical reaction at a low scale to the physical response both at low and high scales. Each of the five separated simulated microstructures of the siliceous limestone can be used as the domain of interest at a microscale to enable deep exploration of the chemical reaction mechanism of ASR based on the methodologies we proposed in [51]. For example, the chemical reaction sequence, the location of the ASR products or the initial expansion sites (inside or outside the aggregate), or the influence of silica fraction on ASR, and so forth, can be clarified as realistically as possible. Moreover, the fracture process at microscale (μm) can be simulated once the information about the ASR products are obtained. Based on the multiscale fracture modelling technique in [50], by passing the mechanical simulation results at microscale to mesoscale, the cracking process of the concrete cube can be simulated then. In this way, the chemical reaction and cracking mechanism induced by ASR at different scales can be captured as much as possible so that better suggestions for preventing ASRs in newly-built structures can be put forward.

## 7. Conclusions

This paper elaborated how to simulate a realistic 3D microstructure of a siliceous limestone based on the autocorrelation function obtained from image analysis performed on 2D SEM-BSE images of the aggregate. The following concluding marks can be made.

(1). Suitable experimental methods should be chosen to obtain 2D images of the aggregate with a clear outline between particles, considering the particle sizes of the target phase, and suitable binarization methods should be chosen to segment the target phase from the images.
(2). The pore and silica fraction distributions on the 2D SEM-BSE images of the limestone can be fitted by a lognormal distribution, which can be used to select representative images for 3D microstructure simulations.
(3). The simulated microstructures of the siliceous limestone are able to retain the visual characteristics, such as the particle shape and spatial scattering, as well as the statistical characteristics of the air void and quartz silica, such as the fraction, characteristic particle size, and the specific surface area of the parent limestone. However, the pore information below the simulation voxel size (1 μm) is lost during the simulation.
(4). The simulated microstructures can be used to assemble the aggregate at a mesoscale embedded in mortar following the obtained lognormal distribution. The average air void and silica fraction show good consistency with the XRD and MIP results.

## Figures and Tables

**Table 1:** Properties of the aggregate used in this study.

Properties	The Limestone
Surface-dried saturation density (g·cm${}^{-3}$)	2.69
Oven-dried density (g·cm${}^{-3}$)	2.67
Bulk density (g·cm${}^{-3}$)	2.71
Water absorption after 24 h (%)	0.51
Specific gravity	2.69

**Table 2:** Comparison of representative statistical variables between the fitted lognormal distribution and raw data.

Phases	Data Resources	Mean Fraction ${}^{1}$, %	Median Fraction ${}^{2}$, %	Mode Fraction ${}^{3}$, %
Pore	Image analysis	3.94	3.41	3.60
	Fitted lognormal function	3.76	3.32	2.58
Quartz	Image analysis	19	19.27	11.4
	Fitted lognormal function	17.03	15.05	11.35

^1^ The mean fraction value. ^2^ The fraction value separating the higher half from the lower half. ^3^ The global maximum fraction value.

**Table 3:** Mineral mass and volume fraction of the siliceous limestone from XRD results.

	Calcite, %		Dolomite, %		Fluorite, %		Quartz, %	
	Mass	Volume	Mass	Volume	Mass	Volume	Mass	Volume
Sample 1	68.60	78.26	4.19	6.84	2.46	2.54	11.36	12.37
Sample 2	70.66	78.34	7.13	5.53	2.40	2.44	10.80	13.69
Sample 3	71.13	79.51	5.8	4.19	3.32	2.71	12.02	13.58
Average 1	70.13	78.70	5.71	5.52	2.39	2.56	11.39	13.21
Average 2	86.79 ${}^{1}$						13.21 ${}^{2}$	

^1^ The average volume fraction of all phases other than quartz. ^2^ The average volume fraction of the quartz

**Table 4:** Statistical data of the simulated 40×40×40 mm${}^{3}$ cube.

Microstructure ID	Pore & Quartz Fraction, %	Volume Fraction ${}^{1}$, %	Cube Pore Fraction ${}^{2}$, %	Cube Quartz Fraction ${}^{3}$, %	Cube Others Fraction ${}^{4}$, %
1	(0.11 6.48)	1.65	0.65	13.93	85.43
2	(0.51 10.41)	20.33			
3	(0.83 20.51)	6.05			
4	(1.45 32.42)	1.24			
5	(2.77 40.91)	0.78			

${}^{1}$ The volume fraction occupied by each simulated 3D microstructure in the simulated mesoscale concrete cube. ${}^{2}$ The average pore fraction over 1 μm of the cube. ${}^{3}$ The average quartz fraction of the cube. ${}^{4}$ The average other minerals fraction of the cube.

**Table 5:** MIP results of the limestone.

Sample ID	Total Pore Fraction, %	Pore Fraction over 1 μm, %
1	0.23	0.17
2	0.55	0.49
3	0.37	0.37
4	0.79	0.79
5	0.25	0.09
6	2.56	2.35
Average	0.8	0.7

## Data Availability

Data is contained within the article.

## References

[1] Silva F.A., Delgado J.M., Azevedo A.C., Mahfoud T., Khelidj A., Nascimento N., Lima A.G. (2021). Diagnosis and Assessment of Deep Pile Cap Foundation of a Tall Building Affected by Internal Expansion Reactions. Buildings.

[2] Delgado J., Nascimento N., Silva F., Azevedo A. (2021). Diagnostic of concrete samples of pile caps affected by internal swelling reactions. Iran. J. Sci. Technol. Trans. Civ. Eng..

[3] Silva F., Delgado J., Azevedo A., Lira I. (2021). Numerical Analysis of Bottle-Shaped Isolated Struts Concrete Deteriorated by Delayed Ettringite Formation. Iran. J. Sci. Technol. Trans. Civ. Eng..

[4] Stanton T.E. (1942). Expansion of concrete through reaction between cement and aggregate. Trans. Am. Soc. Civ. Eng..

[5] Rajabipour F., Giannini E., Dunant C., Ideker J.H., Thomas M.D. (2015). Alkali–silica reaction: Current understanding of the reaction mechanisms and the knowledge gaps. Cem. Concr. Res..

[6] Ponce J., Batic O.R. (2006). Different manifestations of the alkali-silica reaction in concrete according to the reaction kinetics of the reactive aggregate. Cem. Concr. Res..

[7] Leemann A., Münch B. (2019). The addition of caesium to concrete with alkali-silica reaction: Implications on product identification and recognition of the reaction sequence. Cem. Concr. Res..

[8] Sanchez L., Fournier B., Jolin M., Duchesne J. (2015). Reliable quantification of AAR damage through assessment of the damage rating index (DRI). Cem. Concr. Res..

[9] Shin J.H., Struble L.J., Kirkpatrick R.J. (2015). Microstructural changes due to alkali-silica reaction during standard mortar test. Materials.

[10] Bažant Z.P., Steffens A. (2000). Mathematical model for kinetics of alkali–silica reaction in concrete. Cem. Concr. Res..

[11] Dunant C.F., Scrivener K.L. (2010). Micro–mechanical modelling of alkali–silica–reaction–induced degradation using the AMIE framework. Cem. Concr. Res..

[12] Çopuroğlu O., Schlangen E. (2007). Modelling of effect of ASR on concrete microstructure. Key Eng. Mater..

[13] Miura T., Multon S., Kawabata Y. (2021). Influence of the distribution of expansive sites in aggregates on microscopic damage caused by alkali-silica reaction: Insights into the mechanical origin of expansion. Cem. Concr. Res..

[14] Cnudde V., Boone M.N. (2013). High-resolution X-ray computed tomography in geosciences: A review of the current technology and applications. Earth-Sci. Rev..

[15] Fernandes J.S., Appoloni C.R., Fernandes C.P. (2012). Determination of the representative elementary volume for the study of sandstones and siltstones by X-ray microtomography. Mater. Res..

[16] De Boever W., Derluyn H., Van Loo D., Van Hoorebeke L., Cnudde V. (2015). Data-fusion of high resolution X-ray CT, SEM and EDS for 3D and pseudo-3D chemical and structural characterization of sandstone. Micron.

[17] Bostanabad R., Zhang Y., Li X., Kearney T., Brinson L.C., Apley D.W., Liu W.K., Chen W. (2018). Computational microstructure characterization and reconstruction: Review of the state-of-the-art techniques. Prog. Mater. Sci..

[18] Wu W., Jiang F. (2013). Simulated annealing reconstruction and characterization of the three-dimensional microstructure of a LiCoO_2_ lithium-ion battery cathode. Mater. Charact..

[19] Kim S.Y., Kim J.S., Lee J.H., Kim J.H., Han T.S. (2021). Comparison of microstructure characterization methods by two-point correlation functions and reconstruction of 3D microstructures using 2D TEM images with high degree of phase clustering. Mater. Charact..

[20] Sumanasooriya M.S., Bentz D.P., Neithalath N. (2010). Planar image-based reconstruction of pervious concrete pore structure and permeability prediction. ACI Mater. J..

[21] Yu F., Sun D., Hu M., Wang J. (2019). Study on the pores characteristics and permeability simulation of pervious concrete based on 2D/3D CT images. Constr. Build. Mater..

[22] Bentz D.P. (1997). Three-dimensional computer simulation of Portland cement hydration and microstructure development. J. Am. Ceram. Soc..

[23] De Schutter G. The detrimental power of alkali silica reaction: Remarkable case study. Proceedings of the Concrete under Severe Conditions: Environment and Loading (CONSEC-2013).

[24] Andreola F., Leonelli C., Romagnoli M. (2000). Techniques Used to Determine Porosity. Am. Ceram. Soc. Bull..

[25] (2000). BS EN 1097-6:2000, Tests for Mechanical and Physical Properties of Aggregates, Determination of Particle Density and Water Absorption.

[26] (1997). BS EN 932-1:1997, Tests for General Properties of Aggregates, Methods for Sampling.

[27] (1999). BS EN 932-2:1999, Tests for General Properties of Aggregates, Methods for Reducing Laboratory Samples.

[28] Beaucour A.L., Hebert R., Noumowé A., Ledésert B., Bodet R. Thermal stability of different siliceous and calcareous aggregates subjected to high temperature. Proceedings of the MATEC Web of Conferences 6.

[29] Razafinjato R.N., Beaucour A.L., Hebert R.L., Ledesert B., Bodet R., Noumowe A. (2016). High temperature behaviour of a wide petrographic range of siliceous and calcareous aggregates for concretes. Constr. Build. Mater..

[30] Washburn E.W. (1921). Note on a method of determining the distribution of pore sizes in a porous material. Proc. Natl. Acad. Sci. USA.

[31] MacKenzie W.S., Adams A.E., Brodie K.H. (2017). Rocks and Minerals in Thin Section: A Colour Atlas.

[32] Fernandes I., dos Anjos Ribeiro M., Broekmans M.A., Sims I. (2016). Petrographic Atlas: Characterisation of Aggregates Regarding Potential Reactivity to Alkalis: RILEM TC 219-ACS Recommended Guidance AAR-1.2, for Use with the RILEM AAR-1.1 Petrographic Examination Method.

[33] Scrivener K.L., Pratt P. Backscattered electron images of polished cement sections in the scanning electron microscope. Proceedings of the 6th International Conference on Cement Microscopy.

[34] Hu C., Li Z. (2015). Property investigation of individual phases in cementitious composites containing silica fume and fly ash. Cem. Concr. Compos..

[35] Edwin R.S., Mushthofa M., Gruyaert E., De Belie N. (2019). Quantitative analysis on porosity of reactive powder concrete based on automated analysis of back-scattered-electron images. Cem. Concr. Compos..

[36] Sahu S., Badger S., Thaulow N., Lee R. (2004). Determination of water–cement ratio of hardened concrete by scanning electron microscopy. Cem. Concr. Compos..

[37] Russ J.C., Dehoff R.T. (2012). Practical Stereology.

[38] Lim J.S. (1989). Two-Dimensional Signal and Image Processing.

[39] Kim H., Ahn E., Cho S., Shin M., Sim S.H. (2017). Comparative analysis of image binarization methods for crack identification in concrete structures. Cem. Concr. Res..

[40] Wong H.S., Head M.K., Buenfeld N.R. (2006). Pore segmentation of cement-based materials from backscattered electron images. Cem. Concr. Res..

[41] Otsu N. (1979). A threshold selection method from gray-level histograms. IEEE Trans. Syst. Man Cybern..

[42] Keys R. (1981). Cubic convolution interpolation for digital image processing. IEEE Trans. Acoust. Speech Signal Process..

[43] Sahoo K., Dhir P.K., Teja P.R.R., Sarkar P., Davis R. (2020). Variability of silica fume concrete and its effect on seismic safety of reinforced concrete buildings. J. Mater. Civ. Eng..

[44] Quiblier J.A. (1984). A new three-dimensional modeling technique for studying porous media. J. Colloid Interface Sci..

[45] Berryman J.G. (1985). Measurement of spatial correlation functions using image processing techniques. J. Appl. Phys..

[46] Berryman J.G., Blair S.C. (1987). Kozeny–Carman relations and image processing methods for estimating Darcy’s constant. J. Appl. Phys..

[47] Anthony J.W. (1990). Handbook of Mineralogy.

[48] Kim J.J., Fan T., Reda Taha M.M. (2011). A homogenization approach for uncertainty quantification of deflection in reinforced concrete beams considering microstructural variability. Struct. Eng. Mech..

[49] Garboczi E.J., Bentz D.P. (1999). Computer simulation and percolation theory applied to concrete. Annual Reviews of Computational Physics VII.

[50] Qian Z.W. (2012). Multiscale Modeling of Fracture Processes in Cementitious Materials. Ph.D. Thesis.

[51] Qiu X., Chen J., Schlangen E., Ye G., De Schutter G. Introduction of a multi-scale chemo-physical simulation model of ASR. Proceedings of the 15th International Congress on the Chemistry of Cement (ICCC).

